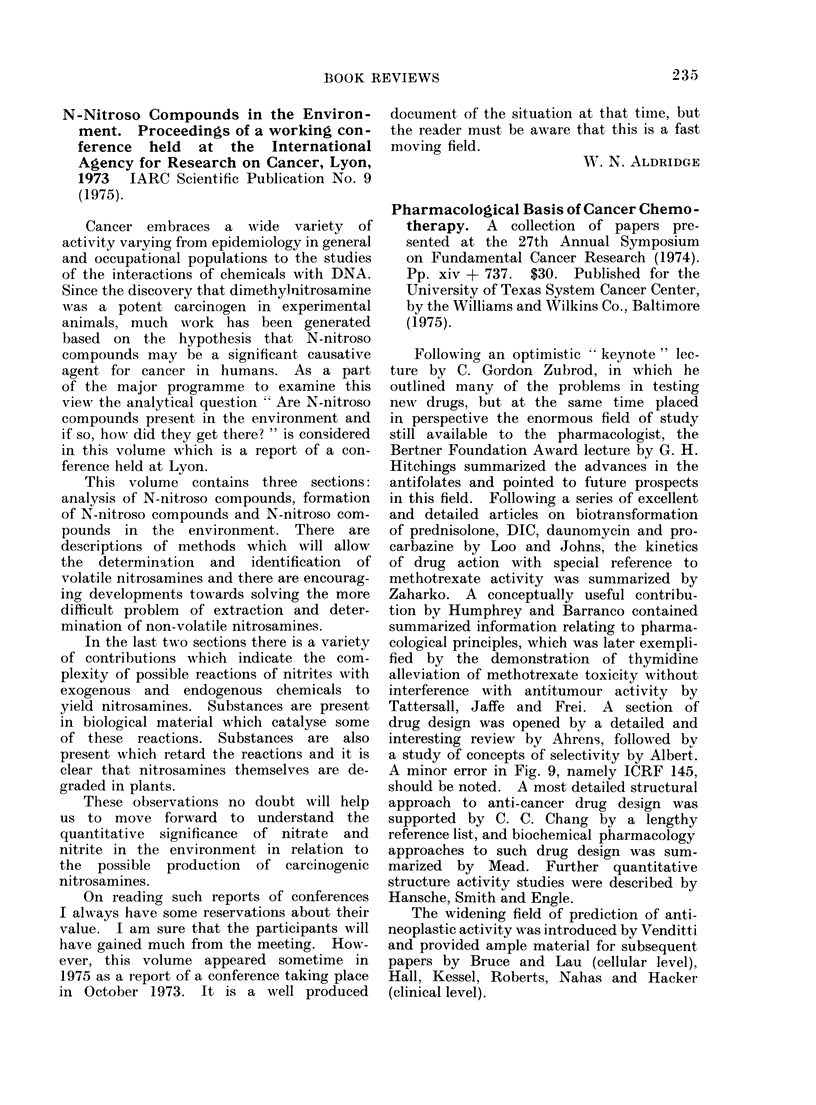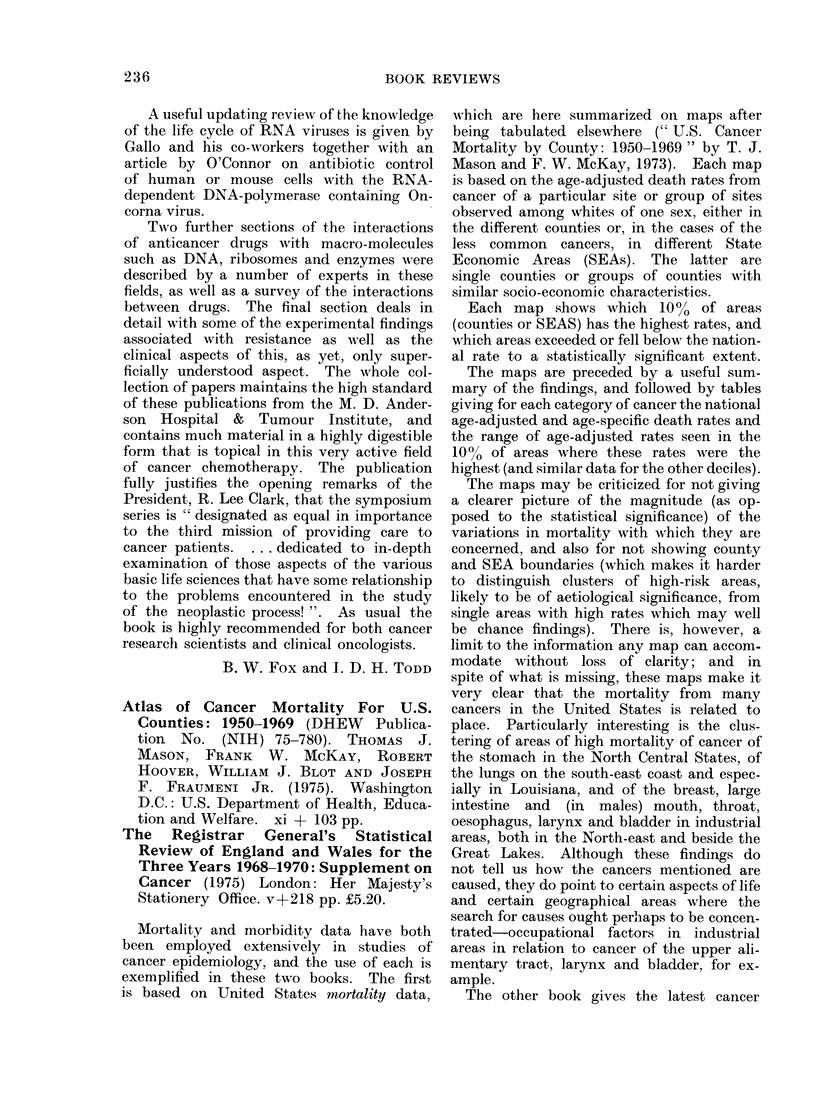# Pharmacological Basis of Cancer Chemotherapy

**Published:** 1976-02

**Authors:** B. W. Fox, I. D. H. Todd


**Pharmacological Basis of Cancer Chemotherapy.** A collection of papers presented at the 27th Annual Symposium on Fundamental Cancer Research (1974). Pp. xiv + 737. $30. Published for the University of Texas System Cancer Center, by the Williams and Wilkins Co., Baltimore (1975).

Following an optimistic " keynote " lecture by C. Gordon Zubrod, in which he outlined many of the problems in testing new drugs, but at the same time placed in perspective the enormous field of study still available to the pharmacologist, the Bertner Foundation Award lecture by G. H. Hitchings summarized the advances in the antifolates and pointed to future prospects in this field. Following a series of excellent and detailed articles on biotransformation of prednisolone, DIC, daunomycin and procarbazine by Loo and Johns, the kinetics of drug action with special reference to methotrexate activity was summarized by Zaharko. A conceptually useful contribution by Humphrey and Barranco contained summarized information relating to pharmacological principles, which was later exemplified by the demonstration of thymidine alleviation of methotrexate toxicity without interference with antitumour activity by Tattersall, Jaffe and Frei. A section of drug design was opened by a detailed and interesting review by Ahrens, followed by a study of concepts of selectivity by Albert. A minor error in Fig. 9, namely ICRF 145, should be noted. A most detailed structural approach to anti-cancer drug design was supported by C. C. Chang by a lengthy reference list, and biochemical pharmacology approaches to such drug design was summarized by Mead. Further quantitative structure activity studies were described by Hansche, Smith and Engle.

The widening field of prediction of antineoplastic activity was introduced by Venditti and provided ample material for subsequent papers by Bruce and Lau (cellular level), Hall, Kessel, Roberts, Nahas and Hacker (clinical level).

A useful updating review of the knowledge of the life cycle of RNA viruses is given by Gallo and his co-workers together with an article by O'Connor on antibiotic control of human or mouse cells with the RNA-dependent DNA-polymerase containing Oncorna virus.

Two further sections of the interactions of anticancer drugs with macro-molecules such as DNA, ribosomes and enzymes were described by a number of experts in these fields, as well as a survey of the interactions between drugs. The final section deals in detail with some of the experimental findings associated with resistance as well as the clinical aspects of this, as yet, only superficially understood aspect. The whole collection of papers maintains the high standard of these publications from the M. D. Anderson Hospital & Tumour Institute, and contains much material in a highly digestible form that is topical in this very active field of cancer chemotherapy. The publication fully justifies the opening remarks of the President, R. Lee Clark, that the symposium series is " designated as equal in importance to the third mission of providing care to cancer patients. ... dedicated to in-depth examination of those aspects of the various basic life sciences that have some relationship to the problems encountered in the study of the neoplastic process! ". As usual the book is highly recommended for both cancer research scientists and clinical oncologists.

B. W. Fox and I. D. H. Todd